# Cell-based therapy in the treatment of musculoskeletal diseases

**DOI:** 10.1093/stcltm/szae049

**Published:** 2024-09-03

**Authors:** Justin Trapana, Jonathan Weinerman, Danny Lee, Anil Sedani, David Constantinescu, Thomas M Best, Francis J Hornicek, Joshua M Hare

**Affiliations:** Department of Orthopaedics, University of Miami Miller School of Medicine, Miami, United States; Interdisciplinary Stem Cell Institute, University of Miami Miller School of Medicine, Miami, United States; Department of Orthopaedics, University of Miami Miller School of Medicine, Miami, United States; Department of Orthopaedics, University of Miami Miller School of Medicine, Miami, United States; Department of Orthopaedics, University of Miami Miller School of Medicine, Miami, United States; Department of Orthopaedics, University of Miami Miller School of Medicine, Miami, United States; Department of Orthopaedics, University of Miami Miller School of Medicine, Miami, United States; Interdisciplinary Stem Cell Institute, University of Miami Miller School of Medicine, Miami, United States; Department of Orthopaedics, University of Miami Miller School of Medicine, Miami, United States; Interdisciplinary Stem Cell Institute, University of Miami Miller School of Medicine, Miami, United States; Interdisciplinary Stem Cell Institute, University of Miami Miller School of Medicine, Miami, United States

**Keywords:** mesenchymal stem cells, musculoskeletal diseases, regenerative medicine, tissue engineering, orthopedic surgery, immunomodulation

## Abstract

A limited number of tissues can spontaneously regenerate following injury, and even fewer can regenerate to a state comparable to mature, healthy adult tissue. Mesenchymal stem cells (MSCs) were first described in the 1960s-1970s by Friedenstein et al as a small population of bone marrow cells with osteogenic potential and abilities to differentiate into chondrocytes. In 1991, Arnold Caplan coined the term “mesenchymal cells” after identifying these cells as a theoretical precursor to bone, cartilage, tendon, ligament, marrow stroma, adipocyte, dermis, muscle, and connective tissues. MSCs are derived from periosteum, fat, and muscle. Another attractive property of MSCs is their immunoregulatory and regenerative properties, which result from crosstalk with their microenvironment and components of the innate immune system. Collectively, these properties make MSCs potentially attractive for various therapeutic purposes. MSCs offer potential in sports medicine, aiding in muscle recovery, meniscal tears, and tendon and ligament injuries. In joint disease, MSCs have the potential for chondrogenesis and reversing the effects of osteoarthritis. MSCs have also demonstrated potential application to the treatment of degenerative disc disease of the cervical, thoracic, and lumbar spine.

## Graphical Abstract

**Graphical Abstract:**
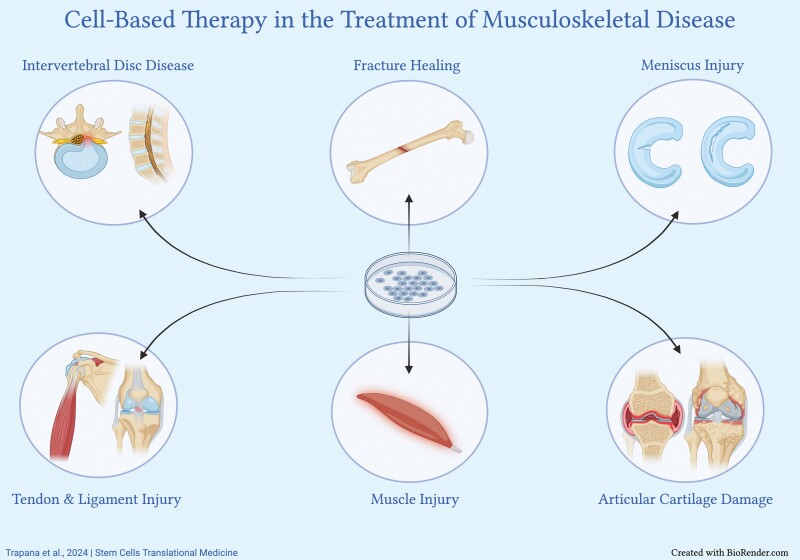

**Significance Statement**

Stem cells have regenerative potential in a variety of tissues. Specifically, mesenchymal stem cells (MSCs) have shown promise for bone and cartilage repair due to their unique immune-modulatory and anti-inflammatory properties. This article reviews the clinical evidence supporting the use of MSCs in orthopedics, outlining their potential for muscle injury and repair, tendon injuries, ligament tears, and mitigating the effects of osteoarthritis. The therapeutic use of stem cells has the potential to revolutionize regenerative medicine.

## Introduction

The past 2 decades have witnessed growing insights into innate regenerative capacity of mammalian adult tissues and the underlying biology of stem cell niches that govern endogenous repair and rejuvenation. The regenerative capacity of tissues and organs ranges substantially from continuous renewal (eg, hair follicles and gut) to extremely limited repair capacity (eg, heart and CNS). As limited tissues have the capacity to regenerate to a completely healthy tissue following injury, there is intense interest in using exogenous cells and cell-derived products to stimulate endogenous repair pathways. Among these strategies, the use of culture-expanded mesenchymal stem/stromal cells (MSCs) has occupied the attention of many scientists and Orthopedic clinicians resulting in extensive research aimed at defining the potential of these cells and related products for cartilage and bone repair.^1^

MSCs are a heterogenous subset of multipotent regenerative cells that can be harvested from various sources including bone marrow (BM), amniotic fluid, placenta, adipose tissue, joint synovium, synovial fluid, dental pulp, endosteum, and periosteum.^2^ MSCs were first described by Friedenstein in the late 1960s to early 1970s as a small population of BM cells characterized by osteogenic potential, adherence to plastic, and the ability to form new, fibroblast-like progeny over many culture passages.^3,4^Friedenstein found that these cells could differentiate in vitro not only into osteocytes but also into chondrocytes and adipocytes. Subsequently, Caplan et al in 1991 termed these cells “mesenchymal stem cells” after discovering their ability to differentiate into more than one type of cell that formed connective tissue in many organs and had many different phenotypic endpoints of differentiation. Caplan pioneered the differentiation of the MSCs into bone-forming osteoblasts and cartilage-forming chondrocytes and described the ideal culture medium and conditions for each.^5^

Growing interest in MSCs led to controversy regarding the nomenclature and criteria for defining populations of cells as MSCs. To address this, the International Society for Cellular Therapy (ISCT) published its position specifying the criteria defining the population of MSCs and recommending the use of the name multipotent mesenchymal stromal cells; however, mesenchymal stem cells remain the most popular name. The ISCT’s published criteria described 3 major requirements: (1) cells must be plastic adherent when maintained under standard culture conditions; (2) cells must express cluster of differentiation (CD)73, CD90, and CD 105 markers and should not express CD34, CD45, CD14, human leukocyte antigen complex (HLA)-DR, CD11b or CD19; (3) cells must possess the ability to differentiate into osteoblasts, chondroblasts, and adipocytes in vitro.^6,7^

One potential conflict with these criteria is the relationship between MSCs and vascular pericytes. Pericytes are contractile cells that line the outer walls of capillaries and other vascular structures. They have similar cell surface markers and characteristics of MSCs and exhibit many similar properties to MSCs when grown ex vivo.^8-11^ For these reasons, some investigators propose that MSCs are derived from pericytes.^12^ However, lineage tracing techniques in transgenic mice reveal that pericytes are not present as local progenitor cells in vivo and are not equivalent to MSCs.^13^ Additional clarification derives from the findings of Khosravi et al^14,15^ using intra-vital microscopy supporting the idea that tissue-resident mesenchymal progenitor cells are localized between capillaries and represent a distinct population from pericytes. Their work suggests that within bony compartments tissue-resident mesenchymal cells are of perivascular origin and can differentiate into functional pericytes in addition to fibroblasts and osteogenic cells.

Also worth noting is that within the bony compartments are different types of endothelial cell (EC)-lined vessels that are selectively surrounded by a perivascular population of cells that are Runx2 and SP7 positive committed osteoprogenitor cells. Endosteal ECs that strongly express CD31/platelet endothelial cell adhesion molecule-1 and endomucin are termed H-type ECs, which are abundantly surrounded by osteoprogenitor cells. These findings confirm the earlier observations of Aubin et al^16^ who described populations of committed and inducible mesenchymal cells in BM. These H-type ECs mediate local growth of vasculature and provide niche signals for perivascular osteoprogenitors, which is important for compromised fracture healing and bone density loss.^17^ The concept of perivascular progenitor cells and their H-type EC microenvironment provides an interesting explanation for loss of bone mass density and a possible therapeutic target to enhance osteogenesis and fracture healing.

To conduct this review, we searched for studies using Pubmed and Data.gov including keywords and headings such as “MSCs, stem cells, muscle, articular cartilage, osteoarthritis, meniscus tear, tendon tear, ligament tear, intervertebral disc, fracture, and trauma.” Preference was given to RTCs conducted since 2017. Animal and preclinical studies were included to demonstrate the biological rational and potential mechanisms underlying the use of MSCs.

### MSC administration

The administration of MSCs is specific to the type of condition being treated along with the intended effect. For most immunological or systemic disorders, intravenous administration has been widely applied whereas for certain types of soft-tissue injuries, MSCs have been directly injected locally into wounds and specific sites. MSCs were originally believed to migrate to sites of injury where they engraft and differentiate into functional tissue cells. However, cell tracking technology as well as long-term follow-up studies have illustrated that this is likely not the case. A high portion of intravenous-infused cells are trapped within organs with large capillary beds such as the lungs and a majority do not migrate to other sites. In addition, the majority of infused cells do not survive more than 24 hours.^18^ As a result, the mechanism of action of MSCs has evolved to the concept that these cells act as trophic mediators to modulate the function of immune cells and resident progenitor cells rather than differentiate into various cell lines.^19^

For the purpose of bone repair, MSCs are usually seeded on transplantable scaffolds such as bioceramics, polymers, and composite biomaterials. Bioceramics such as calcium phosphate and calcium silica demonstrate high bioactivity and biocompatibility but have relatively low toughness and insufficient strength. Polymers such as poly-lactic acid, poly-glycolic acid, and poly-lactic-*co*-glycolic acid demonstrate good plasticity with the ability to support osteoprogenitor cell adhesion and growth. However, they pose disadvantages of weak mechanical properties with risk of plastic deformity and lack of boney integration with provocation of giant cell response. Composite biomaterials typically consist of a composite of ceramics combined with polymers to provide advantages of both scaffolding types and can be tailored for an appropriate balance of strength and toughness.^20^ However, clinical administration and utilization of scaffolding constructs requires further illustration of MSCs’ mechanism of action, standardization of both MSC sourcing, culturing, osteogenic signaling factors as well as scaffold design and composition before further progress can be made toward elucidating the optimal therapeutic approach.

There have been discrepancies between positive results obtained from preclinical animal models and their translation into clinical efficacy in human trials.^21^ Galipeau et al suggested that such differences could arise from the preferential use of fresh MSC preparations in preclinical models compared to cryopreserved in clinical trials. To address this issue, Hoogduijin and Lombardo^22^ performed a concise review of publications using MSCs in experimental animal models of inflammatory diseases and identified methodological details regarding the origin, immunological matching, and status of cells prior to administrations and therapeutic outcomes. Their review suggested xenogeneic MSCs to be equally efficacious for both autologous and allogeneic MSCs in animal models. They cautioned, however, that in other animal models, these results could be opposing such as the case seen in a rat corneal transplantation model in which human MSCs in contrast to rat MSCs failed to prolong allograft survival. Interestingly, freshly cultured allogeneic rat MSCs showed equal efficacy as the same cells after cryopreservation.^23^ Accordingly, Hoogduijin and Lombardo^22^ concluded that MSC administered immediately following thawing retained therapeutic potential equivalent to cultured MSCs in animal models, thereby challenging the disparity between preclinical and clinical therapeutic effects associated with cryopreserved MSCs, fresh MSCs, autologous MSCs, and allogeneic MSCs. These data should be interpreted with some caution as additional studies comparing cultured and thawed MSCs are needed to make definitive conclusions.

With respect to dosing regimens, MSCs are usually administered in rodents at a dose of 50 million cells/kg, while clinical trials in humans range between 1 and 10 million cells/kg. There has been no clear transition made between effective dosing in rodents and humans and data continues to remain limited. In addition, comprehensive studies comparing the effects of single versus repeat dosing at different time points even in animal models are limited.^22^

### Cell sourcing of MSCs

One of the most interesting aspects of MSCs is that their differentiation potential varies based on their tissue of origin. Depending on the specific tissue-derived MSC, these cells display different transcriptomes, proteomes, immunophenotypes, and immunomodulatory activities. As a result, depending on the tissue-specific derived MSC there is a tendency to differentiate into different end-stage lineage cells including osteoblast and chondrocytes.^24^ Bone marrow-derived MSCs (BMD-MSCs) exhibit a superior capacity for osteogenesis and chondrogenesis while; synovial MSCs (S-MSCs) demonstrate greater chondrogenic and proliferation potential than adipose-derived MSCS (AD-MSCs), BMD-MSCs, and MSCs derived from periosteum, fat, and muscle.^25-27^ Umbilical cord blood-derived MSCs (UCB-MSCs) were found to have the highest rate of cell proliferation and clonality and significantly lower expression of markers for senescence compared to BMD-MSCs and AD-MSCs.^28^

The frequency of successful collection and primary frequency of cells harvested from cord blood (CB) are typically low as described by Kögler et al.^29^ However, controversy exists regarding the superiority of certain extraction protocols and harvest compartments for CB. Typically, MSCs are sampled from CB via needle passage inserted into Wharton’s jelly, which does represent a rich population of MSCs.^30^ Still controversial, it is believed that the low frequency of cells harvested from CB is a result of harvesting technique and not a lack of abundance of cells present. Although there is low primary frequency in CB, these cells can be expanded to at least 10^15^ cells while maintaining a normal karyotype. Schugar et al^31^ suggest that the most abundant source of MSCs arises from the perivascular zones of human umbilical cord (UC) and propose a technique of digesting the UC with collagenase so as to select against hematopoietic and endothelial cells while maximizing yields of cells expressing CD146 and MSC markers. Sarugaser et al^32^ using a colony forming unit frequency (CFU-F) assay as a surrogate MSC measure demonstrated a CFU-F of 1:300 at harvest from an isolated UC perivascular population. In contrast, the CFU-F is estimated to be 1:10 000 to <1:100 000 reported for BMD-MSCs. Also, the population doubling (PD) time of UC-MSCs has been reported to be 24 hours as compared to 40 hours for BMD-MSCs.^33,34^ These differences in proliferative capacities, extraction methods, and differentiation proclivities are significant because the location of MSC extraction has an impact on differentiation potential in recipient tissues and may necessitate the addition of different cytokines or paracrine factors to obtain the desired potential as will be discussed below. In addition, the difference in PD times between specific tissue-derived MSC affects ex vivo culture expansion times and viability of cell banking. Therefore, researchers and clinicians must be mindful of the different tissue-derived MSCs and tailor their choices according to their desired purpose. These differences also highlight how the yield of MSCs can depend on a multitude of tissue, biologic, and manufacturing factors and a growing need for standardization both in research and clinical practice to further advance the field.

### MSC interaction with host cells

The current prevailing theory regarding MSC mechanism of action is that these cells produce a variety of trophic mediators including growth factors, angiogenic factors, and immune regulatory factors that modulate the function of immune, vascular, and progenitor cells.^35-39^ The secretomes of MSCs include extracellular vesicles (EVs) containing microRNA, organelles, and proteins that control cell function. An elegant example is the release of osteoclastic factor receptor activator of nuclear factor kappa-B ligand (RANKL) by MSCs delivered on biomimetic scaffolds. The secretomes containing RANKL serve as a supplement of the cytokine to enhance the formation of tartrate-resistant acid phosphatase positive (TRAP+) cells in bone, yielding a potential treatment for osteopetrosis.^40^ Additional studies demonstrate the potential of cell-free therapies utilizing MSC secretomes. For example, Joseph et al^41^ used immunohistochemistry and Western blot to demonstrate that MSC conditioned culture media contained important growth factors and cytokines including vascular endothelial growth factor (VEGF), transforming growth factor-beta 1 (TGF-β 1), fibroblast growth factor 2, insulin like growth factor 1, stem cell factor, and interleukin-6 (IL-6). This study further demonstrated that allogeneic and xenogeneic application of MSC conditioned culture media significantly improved wound healing with minimal scar formation. As a result, MSC secretomes may be effective cell-free therapies and also provide support for the idea that MSCs exert important bioactivity through paracrine signaling. It is important to note that studies evaluating the introduction of secretome-deficient MSCs evoked similar responses as MSC infusion with secretome-sufficient MSCs. The spike in serum cytokines and chemokines observed from these studies from host cells rather than MSCs implies that the effects attributed to MSC secretomes may have other origins as well or may require heterocellular coupling^35-37,42^ Another possible mechanism is that specific endogenous cells such as H-Type ECs play in coupling angiogenesis and osteoprogenitors in fracture healing. The work of Zhang et al^43^ proposes that type H ECs mediate local angiogenesis and provide niche signals for perivascular osteoprogenitors by particular molecular pathways, which can be targeted for osteogenesis. Type H vascular ECs have been found to secrete Noggin and VEGF, which stimulate proliferation and differentiation of osteoprogenitors to regulate osteogenesis. Their work has also shown that the administration of exogenous platelet-derived growth factor type BB (PDGF-BB) and activation of different signaling pathways in bony defects can promote formation of type H vessels and restoration of bone.^42,43^

### Immunomodulatory effects of MSCs

Another attractive property of MSCs is their immune-modulatory and anti-inflammatory effects. Through their production of soluble factors, MSCs can suppress both the innate and adaptive immune systems by attenuating maturation and capacity for antigen presentation of dendritic cells, inhibiting activation and proliferation of T and B lymphocytes, and reducing cytotoxicity of natural killer (NK) and natural killer T (NKT) cells.^44^ In addition, MSCs lack HLA class II/major histocompatibility complex (MHC) and associated cell surface costimulatory molecules allowing MSCs to evade recognition by the immune system.^45,46^ MSC expression of MHC I allows these cells to evade NK cell-mediated cytotoxicity, and lack of expression of MHC II prevents identification by CD4+ T cells, respectively.^3,12^ However despite previous literature suggesting solely inhibitory effects on immune cells, more recent observational studies emphasize the dynamic role of MSCs through crosstalk with the microenvironment. This is exemplified by MSC communication with the innate immune system to act as immunostimulatory cells partly by upregulating MHC class II expression when there are lower levels of interferon gamma.^47^ Therefore, MSCs balance immunosuppression and immunoactivation effects depending on their differentiation state and the host inflammatory microenvironment. This is apparent during fracture healing as MSCs crosstalk with macrophages resulting in downregulation of proinflammatory M1 macrophages and promotion of anti-inflammatory M2 macrophages to support bone formation.^47^

A concern with MSC therapies is the controversial risk of tumor formation.^48^ In this regard, several studies, mostly conducted in rodents or in vitro, demonstrate the capacity for MSCs to contribute to the progression of certain tumor cells. MSCs migrate toward inflammatory sites and incorporate into tumors.^46,48^ Importantly, this concern has not been borne out in clinical evaluation. For example, Hernigou et al^48^ examined 1873 patients treated with BM-derived concentrated cells and monitored for cancer incidence both at the treatment site and systemic locations over a 12.5-year period. Results demonstrated a cancer incidence below that expected in the general population leading to the conclusion that there is no increase in cancer risk in patients after the application of autologous cell-based therapy using BM-derived stromal progenitor cells.^22^ In addition, Lalu et al^49^ performed a systematic review and meta-analysis of prospective clinical trials of intravascular delivery of MSCs in adult and mixed adult/pediatric populations to further explore the safety of MSCs. Outcome measures included acute infusion toxicity, fever, organ system complications, infection, and longer-term adverse events including death and malignancy. A total of 8 RCTs with 321 participants were included. There was no difference in incidence of acute infusion toxicity, organ system complications, infection, death, or malignancy between controls and those that received MSC infusions. The results support the safety of MSCs not only with regards to tumor formation but also for other adverse events. More recently, a phase 1 study assessed safety and cancer-homing ability of allogenic BMD-MSCs in men with localized prostate cancer to determine an ideal cell-based therapeutic vector for delivery of cytotoxic agents. Seven patients received allogenic BMD-MSCs intravenous infusions 4-6 days prior to radical prostatectomy. Pathologic assessment of the prostates demonstrated no detectable MSCs; however, the high rate of lung sequestration of infused MSCs led to early termination of the trial due to perceived futility.^50^ Importantly, the study demonstrated that infusion of MSCs was safe in males with prostate cancer and there was no evidence for MSC homing to the primary malignancy. Although there remains controversy regarding the increased risk of cancer with MSC therapies, and there are preclinical studies to suggest homing of MSCs to sites of primary and metastatic tumor site, clinical data suggest that MSC application in human subjects is safe and poses a limited risk of tumor formation as described above.

The remainder of this review will discuss current and potential applications of MSCs in the treatment of musculoskeletal (MSK) diseases including fracture management, muscle injury and repair, articular cartilage, meniscus pathology, tendon injury, ligament repair, and intervertebral disc applications.

## MSCs application in fracture healing

Fracture healing occurs as a result of a complex sequence of events that often results in complete bone repair to a pre-injury condition. A key feature of fracture healing proceeds through a combination of endochondral and intramembranous ossification.^12^ Intramembranous ossification occurs when bone formation ensues without a cartilaginous phase and the majority of cells that contribute are present in the inner osteogenic layer of the periosteum and endosteum. Ossification begins with the condensation of MSCs producing a collagen-proteoglycan matrix that can bind calcium and become mineralized. A BM cavity is formed and calcified bone spicules become surrounded by compact MSCs that form the periosteum and endosteum. Osteogenesis is made possible due to the presence of MSCs that form the inner sheet of the periosteum and the endosteum.^51,52^

Endochondral ossification occurs when an initial synthesis of cartilaginous scaffolding is followed by its calcification.^53^ This calcified scaffold is then resorbed by osteoclasts, which permits vascular invasion and osteogenesis to occur. This process of angiogenesis after a fracture is stimulated by hematoma formation and a subsequent series of intercellular connections and release of growth factors, cytokines, and interleukins that cause chemotaxis to the fracture site. Angiogenesis is crucial for trafficking of MSCs from the systemic circulation and also from previously described perivascular osteoprogenitor cells to the fracture site.^51,54^ In addition, H-type ECs mediate local growth of the vasculature and provide a niche signal for perivascular osteoprogenitors.^16,17^

Capitalizing on these events, BM extracts have been used for treating nonunion fractures with interest in specific molecular pathways to boost H-type vessel formation and osteogenesis associated with compromised fracture healing or bone mass loss.^55^ Two factors that have been found to act synergistically to promote MSCs migration to fracture sites and fracture site repair are stromal cell-derived factor (SDF)-1 and monocyte chemotactic protein-3.^56^ Others have noted that the expression of bone morphogenic protein-2 and (SDF)-1 increase MSC migration and differentiation to stimulate fracture healing. SDF-1 promotes the autocrine release of VEGF and basic fibroblast growth factor (bFGF) paracrine factor which are both associated with inhibiting cellular apoptosis.^56^ It is important to note that VEGF also plays an essential role in coupling vascular and skeletal morphogenesis. VEGF stimulates the vascular tip as well as bone-forming progenitors along with helping to incite mobilization of nutrition, oxygen, and minerals necessary for osteogenesis. Release of VEGF from MSCs not only plays a role in necessary angiogenesis before osteogenesis but also influences lineage fate between osteoblasts, chondrocytes, or adipocytes.^57^ Also of note, platelets release TGF-β in the inflammatory phase of fracture healing which in turn participates in callus formation and effective chemotaxis of BMD-MSCs.^58^

**Figure 1.**
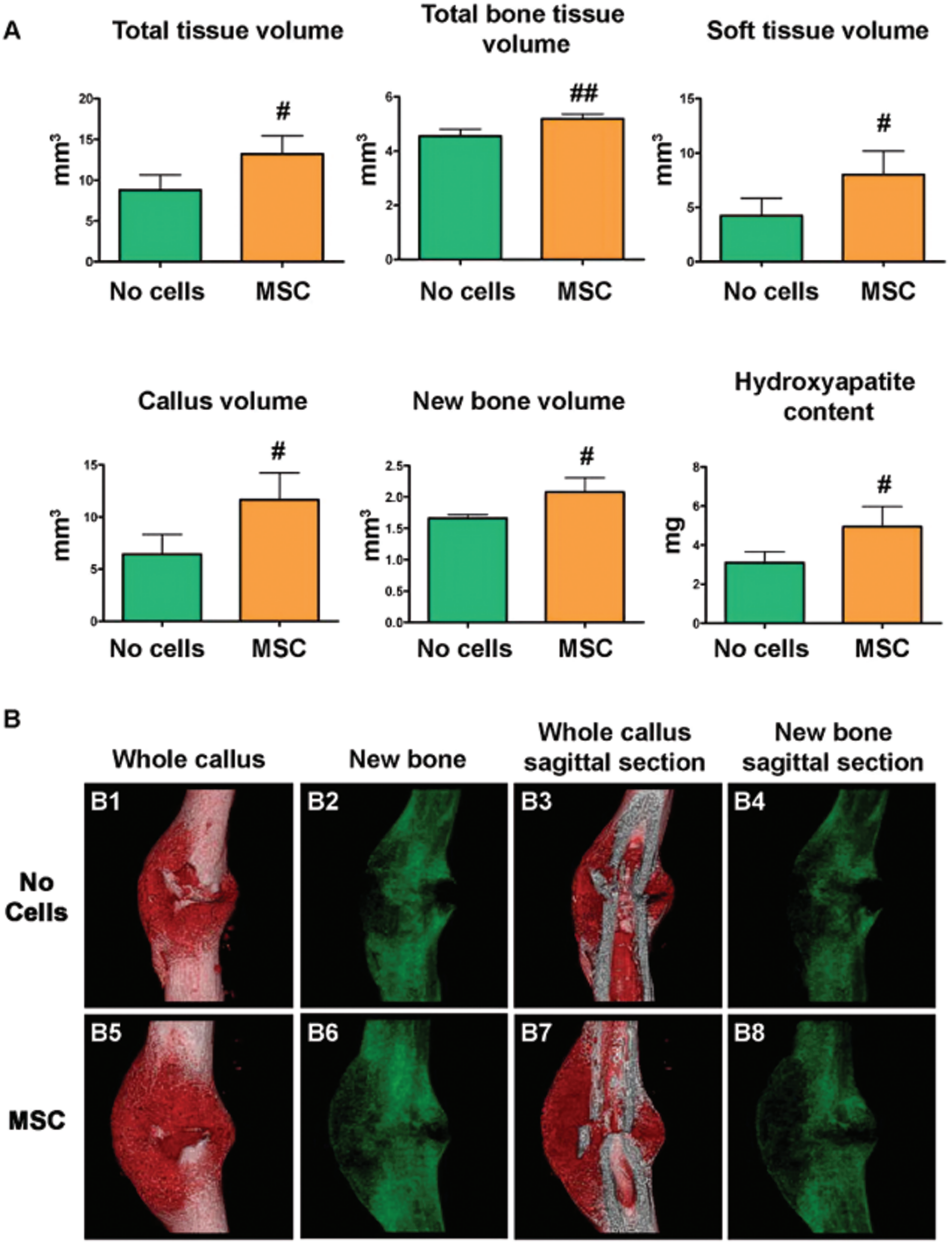
**MSC transplant increases callus size and changes callus morphology in fracture healing sites.** (A) Depicts a μCT analysis performed 14 days after fracture calluses dissected from mice that received MSC transplant compared to controls with no transplant. Callous and new bone volume was calculated after subtracting the cortical bone volume from the total volume and the total bone tissue volume. ^#^*P* < .05 versus no cells; ^##^*P* < .01 versus no cells by Student’s *t*-test. No cells, *n* = 3; MSC, *n* = 6. (B) Three-dimensional reconstruction of whole calluses (B1, B2, B5, and B6) and sagittal sections (B3, B4, B7, and B8) were obtained 14 days after tibial fracture in calluses from mice that were transplanted with MSC or control untransplanted (no cells).^51^ Figure 1 and caption reprinted from Granero-Moltó et al.^51^ Copyright 2009 by Oxford University Press. Reprinted with permission.

Understanding the multiple pathways involved in MSC regenerative abilities in bone osteogenesis is important in allowing the design of novel MSC-based therapies to treat fractures. For example, Granero-Moltó et al^51^ used a stabilized tibia fracture mouse model to determine the dynamic migration of transplanted MSCs to the fracture site and engraftment within the callus endosteum. In addition, they clarified MSCs’ contribution to the repair process and their role in modulating the injury-related inflammatory response (Figure 1). Building on these concepts, Wang et al^59^ used a C57 murine unilateral, transverse femur fracture model to determine the optimal time to inject BMD-MSCs for fracture healing. Their study had multiple implications: (1) injection of BMD-SCs improved bone healing, (2) injection of BMD-SCs 7 days postfracture promoted accelerated fracture healing and callus/bone quality, (3) the SDF-1-chemokine receptor 4 (CXCR) pathway played a key role in the fracture healing process, and (4) MSCs contributed therapeutically to delayed fracture healing and nonunions. Moreover, Emadedin et al^60^ demonstrated the safety of percutaneous autologous BMD-MSC implantation for the reconstruction of human lower limb long bone atrophic nonunion. However, it was noted that further double-blind, controlled clinical trials are necessary to assess the efficacy of this treatment. Recently, Mott et al^61^ preformed a systematic review of 94 studies assessing the evidence for the use of stem cells in fracture healing. The authors found that there was insufficient high-quality evidence to determine the efficacy of stem cells for fracture healing with lack of technique standardization cited as their major limitation. Also, the heterogeneity of assessment of fracture consolidations along with the lack of uniformity in outcome measurements creates significant obstacles to formulating control subjects and measuring outcomes.^62^ To date, there are no registered clinical trials with published data on MSCs and fracture or bone healing. However, in the last 10 years, there are at least 8 clinical trials registered with respect to fracture healing or treatment of nonunion with mesenchymal stem cells. Most trials focus on targeting BMD-MSCs as the primary mesenchymal stem cell treatment for fracture healing (Table 1).^63–70^ One trial is randomizing 32 patients, aged 70-85, to treatment with either endomedullary nailing + BMD-MSC treatment or isolated endomedullary nailing. These patients will be followed for 1 year, primarily assessing safety, CT and X-ray, clinical status, and quality of life.^66^ Improvement in clinical status and quality of life for these patients could have a major clinical impact due to the high levels of morbidity and mortality associated with hip fractures in older individuals (Table 1).^71^

**Table 1.** Recent clinical trials on MSCs in fracture healing.

Title	Cell type	Year initiated/reported	CT.gov ID
Autologous bone marrow concentration for femoral shaft fracture union	Auto-BMD-MSC	2019	NCT03794622
A comparative study of 2 doses of BM autologous H-MSC+biomaterial vs iliac crest autograft for bone healing in non-union (ORTHOUNION)	Auto-BMD-MSC	2017	NCT03325504
Autologous BM-MSC transplantation in combination with platelet lysate (PL) for nonunion treatment	Auto-BMD-MSC	2015	NCT02448849
Allogeneic mesenchymal stromal cells in elderly patients with hip fracture	Allo-BMD-MSC	2015	NCT02630836
Mesenchymal stromal cells for the treatment of non-union fractures of long bones	Auto-BMD-MSC	2014	NCT02230514
Allogenic mesenchymal stem cell for bone defect or non-union fracture (AMSC)	Allo-UCB-MSC, BMD-MSC, AD-MSC	2014	NCT02307435
Allogeneic mesenchymal stem cell transplantation in tibial closed diaphyseal fractures	Allo-AD-MSC	2014	NCT02140528
Evaluation the treatment of nonunion of long bone fracture of lower extremities (femur and tibia) using mononuclear stem cells from the iliac wing within a 3-D tissue engineered scaffold	Auto-BMD-MSC	2013	NCT01958502

## MSCs application in muscle injury and repair

Following muscle fiber injury, the nuclei of damaged fibers undergo apoptosis. Failure of the replacement of these nuclei partially explains the muscle atrophy and weakness that patients experience after moderate or severe skeletal muscle damage.^72^ The endogenous repair mechanism for this injury derives from skeletal muscle stem cells called satellite cells (also called myosatellite cells), which are activated by injury, migrate to the site of injury, and fuse with injured fibers to repopulate the lost nuclei.^72^ Deploying satellite cells as a therapy represents a possible strategy for the treatment of muscle injuries. However, in vitro expansion of these cells has proven difficult and they exhibit limited post-transplant survival.^12^

As an alternative, BMD-MSCs have been explored as a possible therapeutic strategy. In multiple studies conducted in mice, BMD-MSCs were incorporated into myofibrils of injured muscles and gave rise to Pax7+ satellite cells, a marker for muscle regeneration.^73,74^ Furthermore, engrafted MSCs survived repeated cycles of damage without additional transplant with 89% efficiency.^74^ Stromal cell-derived factor 1 SDF-1/CXCR4 signaling and chemokine receptor (CCR2) are implicated in BM cell homing to muscle injury sites.^73^ A study examining the role of CCR2 on muscle regeneration and BM-derived cells found that mice lacking CCR2 demonstrated fewer BM-derived cells and macrophages along with decreased muscle regeneration.^75^ Klimczak et al^76^ hypothesized that the delivery of MSCs into dystrophic muscle will create a microenvironment/ niche supporting homing of myogenic precursor and enhance tropism of stem cells of myogenic origin with CXCR4+ expression to injured muscle expressing SDF-1. These investigators found that MSCs were viable in injured muscle and contributed to regeneration supporting the importance of microenvironment/niche in MSCs muscle therapy. Iyer et al^77^ also demonstrated that exosomes derived from MSCs facilitate recovery of muscle strain injury in small animal models via reduced expression of TGF-β. They concluded that this resulted from modulation of inflammation, fibrosis, and myogenesis. Large animal model studies demonstrated that MSCs can be used to prevent fatty atrophy that can occur with muscle injury. Flück et al^78^ demonstrated molecular, microscopic, and macroscopic evidence of the prevention of fatty atrophy of detached rotator cuff muscle after tendon repair in an ovine model. They concluded that injection of micro-tissues from MSCs result in a paracrine cascade of events results in expansion of extracellular ground substance, increased tissue water content, and introduction of tenascin C-associated myogenic reactions. Coupled with downregulation of differentiation marker peroxisome proliferator-activated receptor gamma ultimately leads to prevention of fatty atrophy in repaired infraspinatus detachment in sheep models.

At present, there are no clinical trials registered to investigate the effect of MSCs on muscle healing, as all trials focus on the myotendinous junction or the muscle-tendon unit, rather than isolation of the muscle itself. Current studies are also exploring MSC use in the treatment of various dystrophic diseases or spastic palsies.^79–82^ However, there is ongoing research investigating the potential role for MSCs in frailty patients to help mitigate sarcopenia, dynapenia and loss of strength with aging. Clinical trials in this area have demonstrated some potential in the CRATUS phase 1 and 2 trials exploring the concept of rejuvenating aged niches and recapitulating their bioactivity using allogenic BMD-MSC in patients with mild frailty. The results of these trials suggest evidence of bioactivity and effectiveness of BMD-MSCs to combat sarcopenia and age-related muscle wasting with improved hand grip strength, increased 6-minute walk distance and reduced circulating TNF-α (inflammatory cytokine linked to frailty related sarcopenia and loss of strength with aging).^83–87^ In turn, based upon these promising findings, future clinical trials dedicated to orthopedic muscle injury are warranted to identify treatment targets for muscle strains, traumatic lacerations, and other injuries to muscle belly tissue.

## MSCs application in articular cartilage disease and osteoarthritis

An estimated 30.8 million adults in the United States and in excess of 300 million worldwide suffer from osteoarthritis (OA).^88^ Articular cartilage is the hyaline cartilage at the end of long bones and comprises a thin layer bonded to the perforated cortical bone adjacent to intervertebral discs that provides a low friction surface for articulation and transmission of weight between joints.^88,89^ Although the half-life of collagen within cartilage is long, articular cartilage heals very slowly and lesions are difficult to manage because of tissue biochemical properties, acellularity, and limited self-renewal potential.^12,88^ Moreover, OA is not solely a wear and tear disease but represents a complex process that includes inflammatory and metabolic factors such as metalloproteases and inflammatory cytokines.^88^ In addition, OA affects not just the articular cartilage but the entire joint and surrounding soft tissues particularly the synovium, infrapatellar fat pad, and subchondral bone.^88^

Subchondral bone is the layer of bone beneath the hyaline cartilage and cement line, divided anatomically into the subchondral bone plate and subchondral bone trabeculae. It provides mechanical and nutritional support for cartilage and changes in its microenvironment can impact cartilage metabolism.^90^ The synovium is a specialized tissue lining diarthrodial joints, responsible for maintaining synovial fluid volume and composition to aid in chondrocyte nutrition, and has an outer layer rich in collagen and a relatively acellular inner layer dominated by fibroblasts as well as macrophages. It is important to note that synovial fluid facing macrophages are master regulators of joint homeostasis and play an essential role in the changes that lead to osteoarthritis and other joint inflammation.^91^ In osteoarthritis, the synovium undergoes histological changes, characterized by synovial lining hyperplasia, fibrosis, and stromal vascularization, which leads to macrophage infiltration and subsequent cytokine production, angiogenesis, and cartilage degradation.^92^ The secretome of MSCs, which includes growth factors, cytokines, chemokines, and lipids, among others, plays a crucial role in their therapeutic properties. These paracrine factors act on the microenvironment to influence the phenotype of target cells such as promoting anti-inflammatory M2 macrophage polarization.^93,94^ Extracellular vesicles (EVs), such as microvesicles or exosomes, are a key part of the secretome and facilitate communication between different cell types and prior in vitro studies suggest immunomodulatory, regenerative, anti-catabolic, and chondroprotective properties.^95^ EVs are an attractive cell-free therapy because they provide the same clinical benefits without the potential risks of live cell infusions. In addition, EVs show promise in the treatment of OA by reactivating aging MSCs; however, the main clinical challenge is the low production of exosomes.^93^

Given the multitude of tissues affected and the involvement of different inflammatory pathways, the proposed versatility of MSCs have emerged as a potential therapeutic option in the treatment of OA. Several clinical studies have examined the possible benefits of MSCs for OA. A variety of delivery methods and cell processing protocols have been deployed, with encouraging results along with at least one approved product for clinical use.^96–118^

MSCs have shown promising clinical benefits in patients with osteochondral defects. Bashir et al^96^ reviewed 4 clinical trials, testing MSCs injection, that demonstrated improved visual analog scale (VAS) pain scores, reduced cartilage defect as seen on magnetic resonance imaging (MRI), and improved functional measures such as functional rating index and ability to climb stairs. A systematic review by Migliorini et al^97^ of MSC injections for knee OA including 18 studies comprising 1069 treated knees tested the impact of treatment on a variety of clinical outcomes, including the VAS, Western Ontario and McMaster Universities Osteoarthritis Index (WOMAC) score, mean walking distance, Lequesne scale, and Knee Injury and Osteoarthritis Outcome (KOOS) score at 6- and 12-month follow-up. This analysis supported the conclusion that MSC injections for knee OA represent a feasible therapy producing a meaningful improvement of all clinical and functional outcomes, regardless of cell source. Most notably, mean walking distance improved from 71.90 to 152.22 and 316.72 m at 6- and 12-month follow-up, respectively. In addition, mean VAS improved from 18.37 to 30.98 and 36.91 at 6- and 12-month follow-up, respectively. However, these results should be taken with caution as many studies do not provide convincing evidence of long-term benefits after 3-5 years.^98^ For example, the meta-analysis performed by Kim et al^99^ examined clinical outcomes of intra-articular injections of MSCs for cartilage repair in knee OA. In their study, they looked at a total of 5 RCTs and found a significant improvement in Lysholm score and WOMAC when MSCs were used in recommended concentrations but no significant difference in cartilage repair when assessed with MRI. They found that despite favorable clinical outcomes there is limited evidence of pain relief and functional improvement in knee OA and that further rigorously conducted, well-powered RCTs with long-term follow-up are required before MSCs can be supported for use for cartilage repair in OA.

In an attempt to provide more clarification on the potential role of MSCs in the treatment of knee OA, Di Zhao et al^100^ performed a systematic review and network meta-analysis evaluating the clinical efficacy of intra-articular injection of MSCs. The study evaluated intra-articular injections of hyaluronic acid (HA), leukocyte-poor platelet-rich plasma (LP-PRP), leukocyte-rich PRP (LR-PRP), BMD-MSCs, and AD-MSCs against a saline placebo with 6- and 12-month follow-up. The analysis included 43 studies and looked at VAS scores and WOMAC pain subscores. The results were discussed in comparison to HA injections and demonstrated that at 6 months AD-MSCs provided the most pain relief and that LP-PRP was the most effective for functional improvement. During the 12-month follow-up, both AD-MSCs and LP-PRP showed potential clinical pain relief effects; however, the most effective improvements of functional improvement was achieved with LP-PRP.

**Figure 2.**
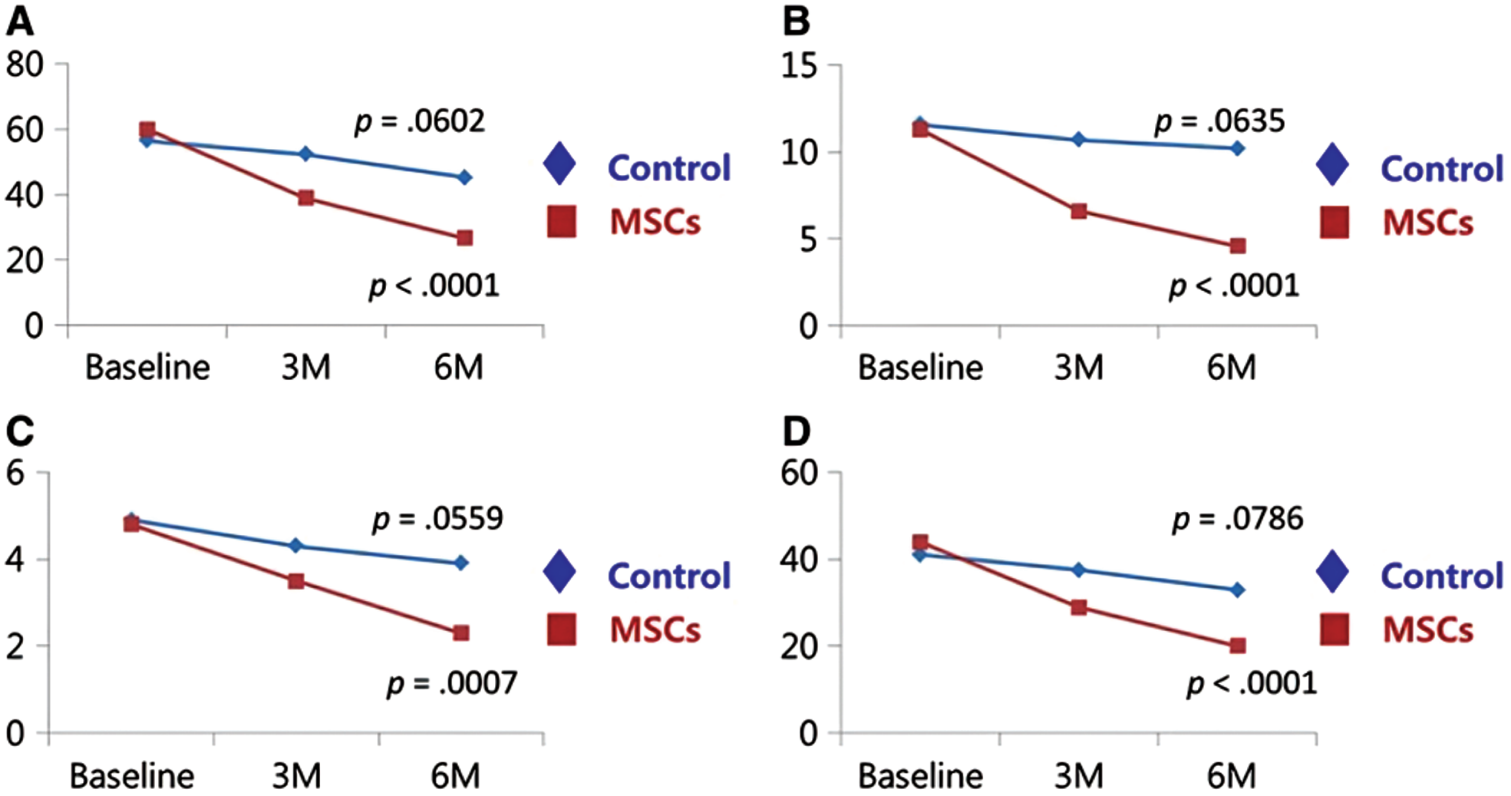
Changes in the WOMAC score during the 6‐month period after intra‐articular injection in the MSC group and control group of OA patients. Patients with injection of AD‐MSC showed significant improvement in the WOMAC score. Patients in the control group did not significantly change in the WOMAC score. (A) The WOMAC total score. (B) The pain subscore of the WOMAC. (C) The stiffness subscore of the WOMAC. (D) The physical function subscore of the WOMAC. Abbreviations: MSC, mesenchymal stem cell; WOMAC, Western Ontario and McMaster Universities Osteoarthritis index.^101^ Figure 2 and caption was reprinted from Lee et al.^101^ Copyright 2009 by Oxford University Press. Reprinted with permission.

Importantly, RCTs continue to emerge supporting the clinical application of MSCs in the treatment of knee OA. A randomized phase 2B placebo-controlled clinical trial conducted that tested the efficacy, safety, and outcome scores of intra-articular injections of autologous AD-MSCs for the treatment of knee OA. AD-MSCs were administered to 12 patients and the group was compared with 12 knees injected with normal saline as a control and were followed for 6 months. The WOMAC score was the primary outcome and with secondary outcomes including radiographic examination and MRI results. The study concluded that a single injection of AD-MSCs led to a significant improvement in the WOMAC score at 6 months compared to the control group. No serious adverse events were observed. MRI evaluation showed no significant changes in cartilage defects at 6 months in the MSC group whereas the defect in the cartilage defects in the control group increased (Figure 2).^101^

There are multiple registered phase 2/3 clinical trials of note showing promising application of MSC in knee OA (Table 2).^102–115^ Of note, 2 clinical trials show promise for improvement in outcomes of pain and/or functional ability after therapy. In addition to the clinical improvements, radiographic assessments of articular cartilage improved with the use of BM-MSCs in the knee.^116,117^ One trial followed 32 patients with knee OA over a span of 12 months assessing pain and patient-reported quality of life after treatment with BMD-MSCs, compared to patients randomized to hyaluronate gel treatment. They found the most improvement in the BM-MSC group at 12 months, consistent with other studies.^12,88,100,106^ Additionally, the 5-year follow-up of a multicenter clinical trial with 114 patients with symptomatic, large, full-thickness defects showed superiority of the combination treatment of allogeneic UCB-MSCs and 4% hyaluronate (UCB-MSC-HA) when compared to microfracture with respect to cartilage grading on second look arthroscopy.^118^

**Table 2.** Recent phase 2 and 3 clinical trials on MSCs in articular cartilage disease and osteoarthritis.

Title	Cell type	Last update posted	Results	Number of patients	CT.gov ID
Allogenic adipose tissue-derived mesenchymal progenitor cells for the treatment of knee osteoarthritis	Allo-AD-MSC	2023	No results available	104	NCT04208646
Effect of mesenchymal stem cells in primary knee osteoarthritis	Auto-AD-MSC	2023	No results available	84	NCT05783154
Multicenter trial of stem cell therapy for osteoarthritis (MILES)	Auto-BMD-MSC, AD-MSC, and UCB-MSC	2023	BM-MSCs improved VAS scores at all timepoints from 1 to 12 points more significantly than AD-MSCs and hUCMSCs. BM-MSCs had comparable VAS scores to cortisteroid injections all timepoints from 1 to 12 months	475	NCT03818737
A phase 3 study to evaluate the efficacy and safety of JointStem in treatment of osteoarthritis	Auto-AD-MSC	2022	Intra-articular injection of autologous culture-expanded AD-MSCs provided significant pain relief and functional improvements in patients with K-L grade 3 osteoarthritis	261	NCT03990805
A phase 2 study to evaluate the efficacy and safety of JointStem in treatment of osteoarthritis	Auto-AD-MSC	2021	Decreased pain, impairment in physical functioning, emotional limitation at 9 and 12 months	28	NCT02674399
Efficacy of allogeneic UCMSCs for treating large defects knee injury	Allo-UCB-MSC	2021	No results available	50	NCT05016011
Chondrochymal for subjects with knee osteoarthritis (knee OA)	Allo-BMD-MSC	2021	No results available	70	NCT05027581
Evaluation of safety and exploratory efficacy of CARTISTEM, a cell therapy product for articular cartilage defects	Allo-UCB-MSC	2021	Higher IKDC score at 12 months	12	NCT01733186
Phase 2B clinical study of chondrogen for treatment of knee osteoarthritis	Allo-UCB-MSC	2020	No results available	100	NCT04520945
Bone marrow versus adipose autologous mesenchymal stem cells for the treatment of knee osteoarthritis	Auto-BMD-MSC and AD-MSC	2020	No results available	54	NCT04351932
Investigation of mesenchymal stem cell therapy for the treatment of osteoarthritis of the knee	Auto-BMD-MSC	2019	Both treatment groups experienced clinically and statistically significant improvement across the KOOS subscales	32	NCT02958267
Clinical trial to evaluate efficacy and safety of JOINTSTEM in patients with degenerative arthritis	Auto-AD-MSC	2019	No results available	24	NCT02658344
Autologous adipose tissue derived mesenchymal stem cells transplantation in patient with degenerative arthritis	Auto-AD-MSC	2019	No results available	18	NCT01300598
Clinical trial to compare ReJoinTM to sodium hyaluronate injection for knee osteoarthritis cartilage defects	Auto-AD-MSC	2018	No results available	28	NCT02855073

There are currently 2 commercially available products available for use outside of the United States that are evaluated in phase 3 trials. JointStem represents an autologous stem cell-based medication injected into the joint cavity of patients with degenerative arthritis for renewal of cartilage along with alleviation of pain and improved joint function at 9- and 12-month time points. The product is currently approved by the Ministry of Health, Labor and Welfare of Japan and is used in a phase 2b/3a double-blinded multicenter trial scheduled for completion on December 30, 2023.^105^ CARTISTEM represents an allogenic UCB-MSC source injected in knee joint cavities and marketed as a treatment of cartilage defects in patients with degrative or repetitive traumatic osteoarthritis. Phase 1/2a clinical trials focusing on the treatment of grade 3-4 articular cartilage defects in knees demonstrated promising IKDC scores at 12 months. The product is currently market-approved for commercial sale by the Ministry of Food & Drug Safety and is currently undergoing phase 3 clinical trial in Japan.^109^

In summary, the totality of evidence demonstrates that MSCs, regardless of source, are safe for clinical use to treat knee OA. There have been a number of RCTs demonstrating the potential promising benefits of MSCs with regards to outcome measures such as the WOMAC, VAS, and mean walking distance. However, some studies still demonstrate some inconsistences with regards to clinical pain relief compared to current PRP injections/standards of practice. For example, a recent phase 2/3 four-arm parallel, multicenter, single-blind, RCT with 480 patients demonstrated non-superiority of autologous BM aspirate concentrate, UC tissue-derived MSCs, PRP, and cortical steroid injections in patients with knee OA after 1 year.^119^ A significant amount of heterogeneity still exists in the literature in regards to outcome, dosing protocols, and dose-dependent effects. In large part, results are short-term demonstrating some improvement over 12 months with few studies showing 5-year follow-up. Important to note that our review preferred more recent studies, for a more comprehensive discussion, Carneiro et al^98^ provides an in-depth review.

## MSCs application in meniscus injuries

The knee meniscus functions to transmit joint reactive forces, lubricate, provide nutrition to the cartilage, and act as a shock absorber.^120^ It is a complex tissue comprised of both cells, specialized extracellular matrix molecules, and region-specific innervation and vascularization. In a healthy adult, there are 3 distinct regions of the meniscus: outer vasculature/neural region (red-red zone), inner completely avascular/aneural region (white-white zone), and a region that separates the two called a red-white region which has attributes of both. The vascularization of this tissue is highly relevant to the regenerative potential of the meniscus with the red-red zone having the greatest potential and the white-white zone having the least.^121^ Current repair techniques for arthroscopic meniscus repair, namely the inside-out, outside-in, and all-inside techniques are effective in treating lesions located in the peripheral vascularized zone; however, satisfactory treatment of the inner avascular region remains a clinical challenge.^121–123^ As a result, there has been a shift toward the utilization of biomaterials and bioengineering for meniscus repair incorporating stem cells and synthetic scaffolds. For example, a study examining the application of nanofiber-based scaffolds seeded with MSCs for the healing of avascular meniscus tears reported that the nanofiber-based scaffolds released collagenase which, enhanced meniscus healing in both in vitro and in vivo settings.^123^

**Figure 3.**
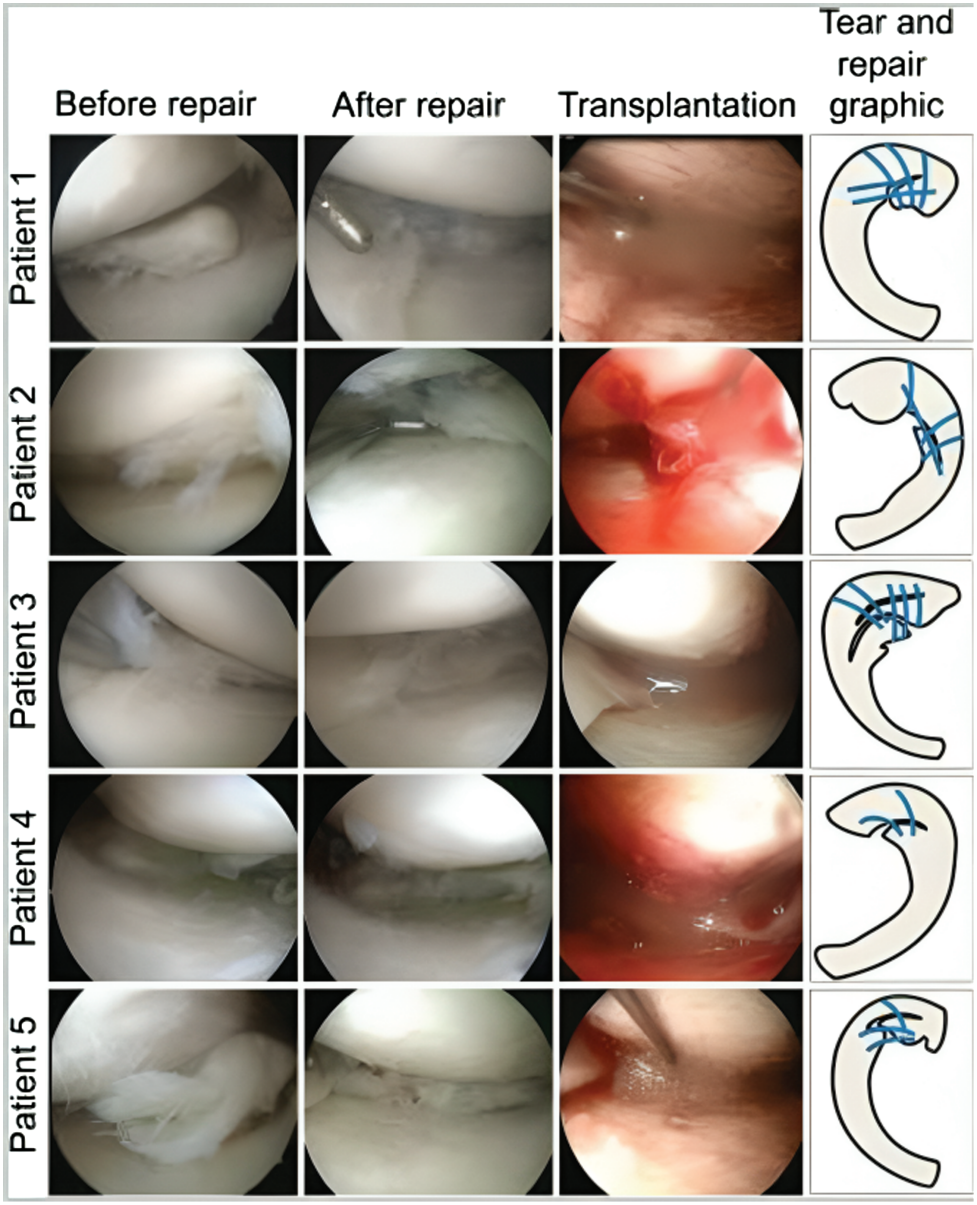
Torn meniscus before and after repair and transplantation of synovial MSCs. Arthroscopic images before and after repair and during transplantation of synovial MSCs, and tear & repair with suture threads (indicated by blue lines) graphics are shown.^125^ Figure 3 and caption reproduced from Sekiya et al.^125^ Copyright by Sage. Reprinted with permission.

Recently, several clinical series employing MSCs have been conducted, including 2 by Sekiya et al^124^. The first combined surgical repair and S-MSCs transplantation. This series studied 5 patients with complex degenerative tears of the medial meniscus. They received surgical repair by standard surgical procedure with all-inside and inside-out repairs and a suspension of in vitro cultured autologous S-MSCs were transplanted into the repair utilizing an 18-gauge needle 2 weeks after surgery (Figure 3). Two years following cell transplantation there was an improvement in scores for “pain,” “daily living,” and “sports activities.”^125^ The second study combined meniscus repair with S-MSC transplantation in 6 patients with degenerative flaps and radial tears of the medial meniscus using the same procedure described above. The investigators performed arthroscopy and Lysholm scores along with the appearance of the repair site. The 4 flap tear patients showed tear zones that were completely stable and smooth in 2 patients and partial restoration in 2 patients at 52 weeks. The radial tear patients were reported to have completely healed at the final follow-up as well. The arthroscopy and Lysholm scores at 1 year were found to be higher compared to preoperative levels in all patients. Although the results of these series are encouraging, it is important to note that these studies lacked control subjects.^126^

There is only one randomized double-blind controlled trial studying the effects of MSC injection in knees post partial medial meniscectomy.^127^ The study consisted of 55 subjects in 3 groups receiving percutaneous injections of allogeneic MSCs: 50 × 10^6^ cells, 150 × 10^6^, and a control group only receiving HA. At 12 months, MRI scans demonstrated a significant increase in meniscal volume in 24% of the patients receiving 50 × 10^6^ cells and 6% receiving 150 × 10^6^ cells. None of the control group patients demonstrated increased meniscal volume. However, the study is limited because MRI was the only outcome measure used and clinical outcomes were not assessed.^127–129^ Whitehouse et al^130^ published a case series describing 5 patients who received BMD-MSCs injected onto a collagen scaffold and sutured into the white-white zone meniscus tears. At 12 months, 3 of the 5 patients demonstrated significant clinical improvement scores and MRI scans demonstrated in situ repair along with reduced abnormal scaffold signals; however, the other 2 patients had repeat tears and failed treatment at the 15-month follow-up.^131^ Despite optimistic results of current human studies, MSC studies in humans with meniscal pathology are still in the early stages and require more studies before a reliable assessment can be made.

With respect to the treatment of meniscal injuries/damage with MSCs, there are currently 3 registered clinical trials in the last 5 years (Table 3).^132–134^ The most recent trial investigates the efficacy of SMCS for degenerative meniscal tears.^132^ Another study from 2019 is evaluating the safety of Human Embryonic Stem Cell (hESC)-derived MSC-like cells for a treatment target in meniscus injury.^133^ The final study is incorporating BMD-MSCs for degenerative meniscal tears. Currently, degenerative disease is the predominant focus of meniscal MSC use, which may translate to traumatic sports injury of the meniscus in the future.^134^

**Table 3.** Recent clinical trials on MSCs in meniscus injuries.

Title	Cell type	Year initiated/reported	CT.gov ID
Clinical efficacy of exosome in degenerative meniscal injury (KNEEXO)	S-MSC (exosomes)	2022	NCT05261360
Safety observation on hESC derived MSC like cell for the meniscus injury	Allo-hESC derived MSC like cell	2019	NCT03839238
Mesenchymal stromal cells for degenerative meniscus injury	Auto-BMD-MSC	2018	NCT02033525

## MSCs application in tendon and ligament injuries

Ligament and tendon injuries are common orthopedic problems affecting approximately 17 million patients a year in the United States. These injuries often result in slow healing that requires lengthy rehabilitation and the formation of scar tissue that requires years of remodeling to achieve functional tissue.^135–137^ More serious injuries with complete ligament and tendon disruption of require operative repair or tissue augmentation. Multiple approaches have been used in reconstructive surgeries for ligaments and tendons such as allografts, autografts, and synthetic biomaterials, but all approaches present challenges including donor-site morbidity, tissue rejection, graft failure, and transmission of infectious diseases.^138^ As a result, alternative methods such as cell-based therapies with MSC or MSC-like cells are being explored as adjuncts to surgical approaches.

The majority of studies regarding tendon and ligament healing have used BMD-MSCs, AD-MSCs, endogenous ligament-derived stem cells, and tendon-derived stem cells (TD-SCs). These studies also investigate a variety of delivery approaches including direct injection with or without collagen gel or tissue-engineered scaffolds seeded with MSCs to provide immediate mechanical support to injuries.^135^ Roth et al^139^ recently described that the therapeutic effects of locally applied MSCs are influenced by the cellular and molecular microenvironment at the injury site, in a bidirectional fashion. Modulating macrophages and promoting M1/M2 macrophage phenotype switch with MSCs plays a crucial role in reducing inflammation in injured tendons and joints. Based on this, it is reasonable to pursue future investigations relating to priming regimes of MSCs and tailoring to specific cell types or ECM components. Animal models investigating application of MSCs for ligament healing have focused on the anterior cruciate ligament (ACL) and generally show promise. Kanaya et al^140^ performed intra-articular injection of BMD-MSCs in Sprague-Dawley rats following surgical partial transection of the ACL. At 4 week follow-up, there was healing tissue within the transection gap compared to the contralateral side that received only ACL transection. Oe et al^141^ demonstrated similar positive outcomes when they demonstrated improved biomechanics and histology after administering MSCs in rats with partial ACL transection compared to saline control. In addition, the equine superficial digital flexor tendon and the human Achilles tendon have demonstrated numerous similarities in structure, function, and injury pathophysiology. Specifically, it has been shown that intralesional injection of MSCs in equine models has produced favorable effects, likely enabled by granulation tissue and the enclosed nature of core lesions that may have provided an appropriate scaffolding opportunity. It is hypothesized that a delivery vehicle may allow for various types of scaffolding or 3D tendon-like tissue constructs may improve stem cell retention at the site of injury.^142^ One RCT demonstrated that AD-MSCs treatment might lead to increased collagen cross-linking of remodeling scar tissue in the equine population.^143^ Another smaller study also showed that AD-MSCs and PRP can be considered a safe and effective strategy for the treatment of superficial digital flexor tendonitis in horses.^144^

**Figure 4.**
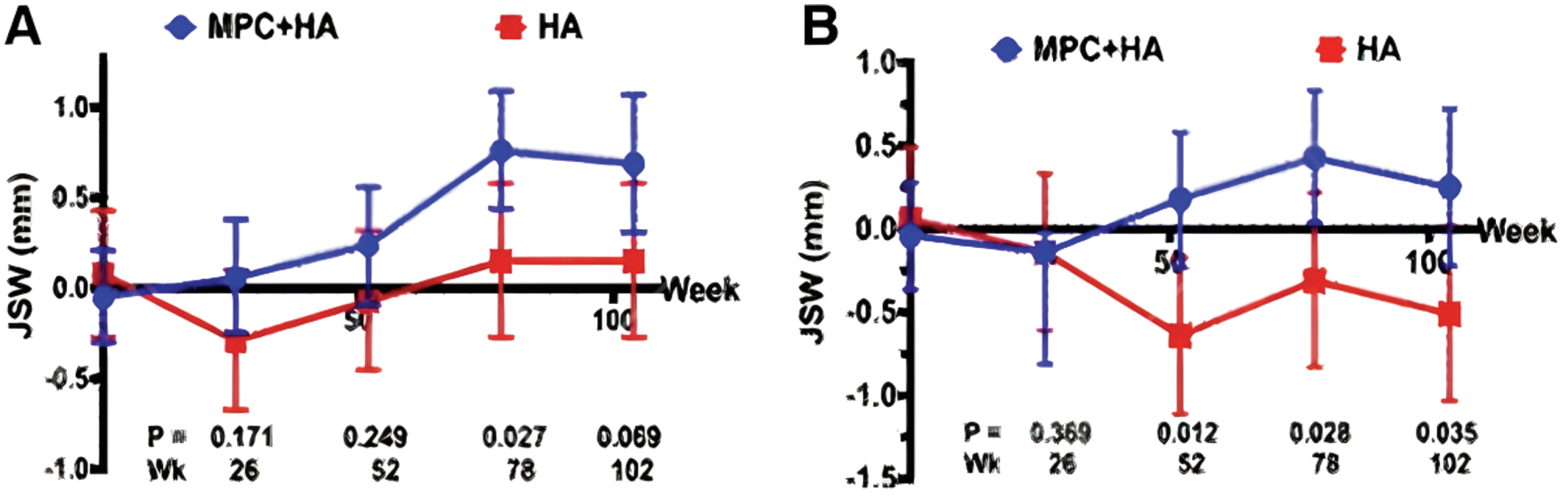
Change from baseline in tibiofemoral joint space width over 24 months (medical joint space width) following ACL reconstruction. (A) Change from baseline in tibiofemoral joint space width over 24 months (medial joint space width. (B) Mean and 95% CI shown. Abbreviations: HA, hyaluronan; MPC, mesenchymal precursor cells.^145^ Figure 4 reproduced from Wang et al.^145^ Copyright by BMC. Reproduced under Creative Commons Attribution (CC-BY) license.

In comparison to the number of animal studies, there is a relative lack of translational human studies involving MSCs and ligament repair. One investigation performed by Wang et al^145^ provides promising evidence evaluating the efficacy of MSC augmented ligament reconstruction. A double-blinded RCT examined 17 patients that received a single intra-articular injection of 75 × 10^6^ allogeneic immature mesenchymal precursor cells (MPCs) in a HA medium coupled with ACL reconstruction. Patients that had associated meniscal injury requiring one-third resection or reconstruction that would alter usual ACL rehabilitation were excluded. At 24-month follow-up, patients who received MSCs demonstrated significantly higher improvements in KOOS and pain scores. In addition, these patients were found to have reduced lateral tibiofemoral joint space narrowing at 24 months (Figure 4). The study did not, however, incorporate measurements of ACL healing such as clinical graft laxity grading or MRI signs demonstrating graft incorporation.^146^

Another study was performed by Moon et al^147^ examining the outcomes of UCB-MSCs in enhancing tendon-graft healing following ACL reconstruction (ACLR). The study enrolled 27 after the exclusion of 3 patients. In the experimental group, UCB-MSCs were suspended in a HA mixture and applied to tendon-bone interfaces of femoral tunnels during ACL reconstruction. A HA only group served as the control. There were no significant differences in the groups when measuring KT-2000 Knee Arthrometer (KT-2000) measurement, pivot shift, or International Knee Documentation Committee (IKDC) scores.

With regards to MSCs and tendon healing, the majority of studies have focused on rotator cuff repair. A study by Gulotta et al^148^ used BMD-MSCs to augment supraspinatus repair compared to supraspinatus repair alone in a rat model. There was no difference in load-to-failure testing at 30 days but a significantly increased strength at the insertion site in animals receiving BMD-MSCs at 45 days. Ina separate study, Hernigou^149^ demonstrated improved healing rates and a lower subsequent tear rate in a case-controlled study that involved applying 51,000 ± 25,000 autogenic BMD-MSCs during arthroscopic full-thickness supraspinatus repair.

Two clinical trials have been completed in the last 5 years employing MSCs in the treatment of tendinous injuries.^150,151^ Another 5 clinical trials are either active, recruiting, or planning to recruit participants to investigate healing using MSCs (Table 4).^152–156^ At this stage, while minimal adverse events have been reported, the literature is not convincing for the use of MSCs in the treatment of tendon or ligamentous injuries.^150,151^

**Table 4.** Recent clinical trials on MSCs in tendon and ligament injuries.

Title	Cell type	Year initiated/reported	Results	Number of patients	CT.gov ID
Treatment of tendon injury using allogenic adipose-derived mesenchymal stem cells (rotator cuff tear)	Allo-AD-MSC	2021	No difference between MSC, control group and placebo in pain, function, or appearance on MR at time points from 6 weeks up to 2 years	24	NCT02298023
Treatment of tendon injury using mesenchymal stem cells (ALLO-ASC)	Allo-AD-MSC	2021	No difference in pain, performance, or appearance on ultrasound	12	NCT01856140
Regeneration of posterior cruciate ligament injury using hypoxic conditioned allogenic adipose mesenchymal stem cell and condition medium	Allo-AD-MSC	2021	No results posted	N/A	NCT04889963
Augmentation of anterior cruciate ligament reconstruction using mesenchymal stem cells and collagen matrix carrier (BioACL)	Auto-MSC (ACL stump)	2020	No results posted	N/A	NCT05582226
Mesenchymal stem cells and amniotic membrane composite for supraspinatus tendon repair augmentation	Allo-AD-MSC	2020	No results posted	N/A	NCT04670302
Treatment of tendon disease using autologous adipose-derived mesenchymal stem cells	Auto-AD-MSC	2018	No results posted	N/A	NCT03279796
Treatment of intractable common extensor tendon injury using mesenchymal stem cells (Allo-ASC)	Allo-AD-MSC	2018	No results posted	N/A	NCT03449082

## Applications of MSCs in treatment of intervertebral disc disease

Degenerative disc disease (DDD) is an umbrella term for intervertebral disc diseases, generally referring to pathologies of the lumbar spine from lumbar disc herniation or other discogenic low back pain. This collection of common diseases that increase with age and are growing in prevalence, has also been a target for treatment with MSCs.^157^ A 2021 meta-analysis of RCTs demonstrated that MSC therapy could decrease VAS scores and Oswestry Disability Index scores, suggesting potential for MSCs as a novel therapeutic intervention for DDD.^158^ The pathological processes of DDD are complex and multifactorial, but are comprised of immunological components involving various IL and tumor necrosis factor-alpha (TNF-α), both of which are implicated in the repair of discogenic disease by stem cells cocultured in an IDD-like environment.^159^

Specifically, at least 5 types of cells have been identified as potential treatment vectors for IDD: BMD-MSCs, AD-MSCs, UCB-MSCs, intervertebral-derived stem cells IDVSCs, and pluripotent stem cells (PSCs).^157^ BMSCs possess the capability to repair degenerated IVD tissues through the promotion of cell proliferation and enhancement of ECM components, including COL I and proteoglycans, while exosomes from BMD-MSCs can increase ECM production and encourage the expression of NP cell-related genes, ultimately achieving the desired effect of IVD self-repair.^160^ AD-MSCs, like BMD-MSCs, have the capacity to differentiate into NP cells, generate distinct growth factors, and contribute to IVD restoration at various levels.^25^ Several studies indicate that AD-MSCs are better suited than BMD-MSCs for the treatment of DDDs due to their superior differentiation potential, but they may also exhibit inferior endochondral osteogenic ability when compared to BMD-MSCs.^161–163^ Like other MSCs, UCB-MSCs are relatively new and have strong proliferation and differentiation abilities, low immunogenicity, and no tumorigenicity.^164–166^ Beeravolu et al^167^ demonstrated that differentiation of UCB-MSCs into chondrogenic progenitor cells in vitro resulted in significant improvements in cell structure, ECM, and glycosaminoglycan content compared to original UCB-MSCs. Furthermore, other studies have indicated that UCB-MSCs promote ECM formation by significantly increasing proteoglycan and COL II expression in the NP.^168^

The applications of IVDSCs face challenges, including low efficiency in separating cartilage endplate stem cells (CESCs) from cartilage endplates (CEPs), difficulty inducing CESCs to differentiate into hyaline cartilage tissue, and a harsh microenvironment of the IVD that poses a challenge to the viability of implanted stem cells.^157^ PSCs are stem cells with high self-renewal, proliferation, and differentiation abilities, and include IPSCs and ESCs. IPSCs are generated by inducing the expression of recombinant transcription factors, while ESCs are derived from early embryos and can differentiate into all 3 germ layers.^169,170^ However carcinogenicity, along with legal and ethical issues have limited the use of PSCs thus far. Presently, the greatest promise for stem cell therapy seems to be with BMD-SCs, AD-MSCs, and UCB-MSCs, with the question of optimal dose, frequency, time, and route of MSC transplantation at different stages of DDD needing to be explored in various clinical trials.^158^

In the last 10 years, there have been 3 completed clinical trials and 3 additional clinical trials currently registered to investigate the treatment of IDD with MSCs. One of which reported results showing the use of BMD-MSCs directly injected into the nucleus pulposus decreased lumbar pain VAS scores at all time points, decreased sciatic pain VAS scores at 6 and 12 months, and increased water content on MRI in lumbar discs at 12 months.^171^ Two other studies are exploring the use of various umbilical MSC types, which could theoretically be superior in restoring disc height in comparison to other cell types due to their actions on the ECM and prior benefits in cell structure.^167,172–176^ Lastly, there is one trial assessing safety of MPCs, which have not been explored as much as other cell types and may provide a new avenue for the stem cell treatment of IDD (Table 5).^174^

**Table 5.** Recent clinical trials on MSCs in intervertebral disc disease.

Title	Cell type	Year initiated/reported	Results	CT.gov ID
Effectiveness and safety of mesenchymal stem cell (MSC) implantation on degenerative discus disease patients (MSC)	Allo-UC-MSC	2020	No results posted	NCT04499105
Human umbilical cord mesenchymal stem cells for the treatment of lumbar disc degeneration disease	Allo-UCB-MSC	2020	No results posted	NCT04414592
Safety and preliminary efficacy study of mesenchymal precursor cells (MPCs) in subjects with lumbar back pain	Allo-MPC	2020	No results posted	NCT01290367
Clinical trial based on the use of mesenchymal stem cells from autologous bone marrow in patients with lumbar intervertebral degenerative disc disease	Auto-BMD-MSC	2017	No results posted	NCT01513694
Human autograft mesenchymal stem cell mediated stabilization of the degenerative lumbar spine	Auto vs allo-“MSCs”	2017	No results posted	NCT02529566
Treatment of degenerative disc disease with allogenic mesenchymal stem cells (MSV) (Disc_allo)	Allo-BMD-MSC	2017	Decreased lumbar pain VAS scores at all time points, decreased sciatic pain VAS scores at 6 and 12 months, and increased water content on MRI in lumbar discs at 12 months	NCT01860417

## Conclusion

The application of cell-based therapies with MSCs in musculoskeletal diseases is a burgeoning and promising area of medicine. MSCs have the potential to provide a diverse and versatile means of treating musculoskeletal diseases. Multiple animal and human models have demonstrated promising results in treating orthopedic injuries/diseases driven largely by the promotion of regeneration of tissues; however, studies to-date have not demonstrated consistent efficacy and further research is required to confirm their safe clinical applications. MSCs can be extracted from a variety of tissues, and biochemical experiments are unraveling the benefits and possible specific roles of specific tissue harvestings. One of the most attractive properties of MSCs is their anti-inflammatory and immunomodulatory effects. However, there are ongoing obstacles to their usage. The majority of studies to-date lack standardization of cell extraction, expansion, preparation, and delivery, which makes a comparison of studies and homogeneous results difficult. In addition, there is ongoing controversy and concern regarding MSCs’ potential risks of tumor formation; however, human trials using MSCs have established their safety in trials so far. Another issue is the relative paucity of human trials, which requires further research to evaluate the safety, efficacy, and applicability of MSCs in orthopedic surgery and the treatment of musculoskeletal diseases. Despite these challenges, progress is being made, and the potential for MSCs to enter the therapeutic armamentarium for musculoskeletal disease remains promising. The application of MSCs has the potential to revolutionize the way we approach and manage musculoskeletal diseases.

## Data Availability

The data underlying this article will be shared on reasonable request to the corresponding author.
